# Divergent Immune and Endothelial Responses to Insulin Resistance in Women with Polycystic Ovary Syndrome

**DOI:** 10.3390/ijms27167473

**Published:** 2026-08-21

**Authors:** Daniela Koleva-Tyutyundzhieva, Maria Ilieva-Gerova, Presiyana Nyagolova, Petya Konsulova, Ekaterina Babadzhanova, Aleksandar Georgiev, Devarshi Kansara, Tanya Deneva, Maria Orbetzova

**Affiliations:** 1 Department of Endocrinology and Metabolic Diseases, Faculty of Medicine, Medical University of Plovdiv, 4002 Plovdiv, Bulgaria; mariyail.ilieva@phd.mu-plovdiv.bg (M.I.-G.); presiyana.nyagolova@mu-plovdiv.bg (P.N.); petya.konsulova@mu-plovdiv.bg (P.K.); ek.babadzhanova@mu-plovdiv.bg (E.B.); maria.orbetzova@mu-plovdiv.bg (M.O.); 2 Department of Diagnostic Imaging, Faculty of Medicine, Medical University of Plovdiv, 4002 Plovdiv, Bulgaria; aleksandar.georgiev@mu-plovdiv.bg; 3 Faculty of Medicine, Medical University of Plovdiv, 4002 Plovdiv, Bulgaria; 22111237@mu-plovdiv.bg; 4 Department of Clinical Laboratory, Faculty of Medicine, Medical University of Plovdiv, 4002 Plovdiv, Bulgaria; tanya.deneva@mu-plovdiv.bg

**Keywords:** polycystic ovary syndrome, insulin resistance, sCD40L, sE-selectin, endothelial dysfunction, immune activation

## Abstract

Polycystic ovary syndrome (PCOS) is a heterogeneous endocrine–metabolic disorder frequently associated with insulin resistance (IR) and increased cardiovascular risk. Soluble CD40 ligand (sCD40L) and soluble E-selectin (sE-selectin) are circulating biomarkers reflecting immune activation and endothelial dysfunction, respectively. However, their differential associations with IR in PCOS, particularly in the context of central obesity, remain incompletely understood. This cross-sectional study included 80 women with PCOS stratified according to waist-to-height ratio (WHtR > 0.50 vs. ≤0.50). Clinical, metabolic, hormonal, inflammatory, and endothelial parameters were evaluated. Correlation and multivariable regression analyses were performed to identify independent determinants of circulating sCD40L and sE-selectin. Women with central obesity exhibited significantly higher fasting insulin, homeostatic model assessment for insulin resistance (HOMA-IR), triglycerides, non-high-density lipoprotein (non-HDL) cholesterol, systolic blood pressure (SBP), and sE-selectin concentrations, together with lower HDL cholesterol. No significant differences were observed in tumor necrosis factor alpha (TNF-α), interleukin-6 (IL-6), or sCD40L. In adjusted regression models, fasting glucose independently predicted sCD40L (β = −0.27, 95% confidence interval (CI): −0.50 to −0.04, *p* = 0.020), whereas fasting insulin emerged as the strongest determinant of sE-selectin (β = 0.41, 95% CI: 0.17 to 0.65, *p* < 0.001). These findings suggest distinct associations of immune and endothelial biomarkers with IR in PCOS. Assessment of sCD40L and sE-selectin may provide complementary information for early cardiometabolic risk stratification in affected women.

## 1. Introduction

Polycystic ovary syndrome (PCOS) represents far more than a reproductive disorder; it is a multifaceted metabolic and endocrine condition that connects gynecologic, metabolic, and cardiovascular domains. Affecting approximately 5–20% of women of reproductive age, PCOS is increasingly recognized as a lifelong disorder with consequences extending beyond menstrual irregularities and infertility [1,2]. The syndrome’s heterogeneity—spanning hyperandrogenism, ovarian dysfunction, and variable metabolic phenotypes—reflects complex interactions among genetic predisposition, environmental factors, and lifestyle influences [3]. Among its metabolic features, insulin resistance (IR) stands out as a central and unifying abnormality, contributing to both hyperandrogenism and heightened cardiometabolic risk [4,5]. Yet, the nature of the link between IR and vascular health in PCOS remains only partially understood.

Mounting evidence suggests that immune dysregulation and endothelial activation form a biological interface between metabolic stress and cardiovascular disease in PCOS [6,7]. Recent evidence has further emphasized the contribution of systemic low-grade chronic inflammation to PCOS, with altered immune-cell activity and dysregulation of inflammatory mediators such as tumor necrosis factor alpha (TNF-α) and interleukin-6 (IL-6) being closely interconnected with obesity, insulin resistance, and metabolic dysfunction [8,9]. At the same time, contemporary evidence highlights the broader cardiovascular burden associated with PCOS, including obesity, impaired glucose metabolism, dyslipidemia, hypertension, and other early vascular abnormalities [10]. These observations support the concept that inflammatory and metabolic disturbances may contribute to early vascular injury before clinically overt cardiovascular disease becomes apparent.

Importantly, recent clinical evidence provides further support for the presence of endothelial dysfunction in women with PCOS. In a 2025 cross-sectional study, women with PCOS demonstrated impaired endothelial function assessed by reactive hyperemia index compared with a normative reproductive-age cohort, even though conventional measures of cardiovascular risk and cellular aging did not differ significantly between groups [11]. Complementary evidence has highlighted an imbalance between vasodilatory, vasoconstrictory, oxidative, and inflammatory pathways as potential contributors to endothelial dysfunction in PCOS [12]. These findings reinforce the importance of identifying circulating biomarkers that may capture early endothelial activation and vascular stress in women with PCOS.

Among these, soluble CD40 ligand (sCD40L) and soluble E-selectin (sE-selectin) are of particular interest due to their dual role as mediators and markers of vascular-immune cross-talk. sCD40L, primarily released by activated platelets and T lymphocytes, is a potent amplifier of inflammation, stimulating endothelial cells to express adhesion molecules, cytokines, and tissue factors [13,14]. Elevated sCD40L levels have been linked to IR, thrombosis, and atherosclerosis in a range of metabolic conditions [15]. Conversely, sE-selectin, a soluble form of the endothelial adhesion molecule E-selectin, reflects direct endothelial activation and facilitates leukocyte rolling and adhesion to the vascular wall [16]. Increased circulating sE-selectin has been reported in obesity, metabolic syndrome, and early vascular dysfunction [17,18]. Importantly, these molecules—though both linked to inflammation—capture distinct biological processes: sCD40L reflecting immune and platelet activation, and sE-selectin denoting endothelial stress. Their combined evaluation could thus provide a nuanced picture of how IR differently affects immune and vascular compartments in PCOS.

Although both biomarkers have been associated with metabolic and cardiovascular abnormalities, their relative and potentially divergent relationships with insulin resistance and central adiposity have not been systematically examined within the same PCOS population.

Central obesity, typically assessed via waist-to-height ratio (WHtR) or waist circumference, further modulates these pathways [19]. The pattern of fat distribution, rather than total adiposity, may critically determine the balance between metabolic inflammation and endothelial integrity [20]. However, whether immune activation (indexed by sCD40L) and endothelial dysfunction (indexed by sE-selectin) are similarly or divergently influenced by IR in PCOS remains unclear.

Therefore, this study aimed to investigate whether biomarkers of immune activation (sCD40L) and endothelial activation (sE-selectin) exhibit differential associations with IR in women with PCOS stratified by central obesity. We hypothesized that these biomarkers would display distinct relationships with metabolic risk factors, reflecting different pathophysiological pathways linking adiposity, IR, and vascular risk in PCOS.

## 2. Results

### 2.1. Age, Anthropometric and Clinical Characteristics According to Central Adiposity

Eighty women with PCOS were included in the study and stratified according to central obesity (waist-to-height ratio, WHtR > 0.50, *n* = 40; WHtR ≤ 0.50, *n* = 40). The two groups were comparable in age (*p* = 0.074). As expected, women with central obesity exhibited significantly higher body weight, body mass index (BMI)), waist circumference, hip circumference, waist-to-hip ratio (WHR), and WHtR values (all *p* < 0.001), confirming marked differences in overall and central adiposity. Systolic blood pressure (SBP) was also higher in the centrally obese group (*p* = 0.004), whereas diastolic blood pressure (DBP) did not differ significantly (Table 1).

Effect size analysis demonstrated large between-group differences in body weight (Cohen’s *d* = 2.6, 95% CI: 2.1–3.1), BMI (*d* = 2.9, 95% CI: 2.4–3.4), waist circumference (*d* = 3.9, 95% CI: 3.3–4.5), WHR (*d* = 2.2, 95% CI: 1.7–2.7), and WHtR (*d* = 3.4, 95% CI: 2.9–4.0). The difference in SBP was moderate (*d* = 0.70, 95% CI: 0.24–1.15), whereas the difference in DBP was small (*d* = 0.44, 95% CI: −0.01–0.89).

### 2.2. Metabolic and Atherogenic Parameters

Central obesity was associated with a more adverse metabolic profile (Table 2). Fasting insulin (IRI 0′) and homeostatic model assessment for insulin resistance (HOMA-IR) were markedly increased in the centrally obese group (*p* < 0.001). Fasting glucose (GLU 0′) showed a modest upward trend but did not reach statistical significance. Serum triglycerides (TG) were significantly higher in women with central obesity (*p* < 0.01), while high-density lipoprotein cholesterol (HDL-C) was reduced (*p* < 0.01). Total cholesterol (TC) and low-density lipoprotein cholesterol (LDL-C) did not differ significantly between groups. Non-high-density lipoprotein cholesterol (non-HDL-C) values were significantly higher in women with central obesity compared with those without (*p* = 0.030), further indicating an atherogenic lipid pattern in this subgroup (Table 2).

Effect size analysis demonstrated large between-group differences in IRI 0’ (Cohen’s *d* = 1.03, 95% CI: 0.56–1.50), HOMA-IR (*d* = 0.94, 95% CI: 0.48–1.41), and TG (*d* = 1.14, 95% CI: 0.66–1.61). HDL-C showed a moderate-to-large effect in the opposite direction (*d* = −0.77, 95% CI: −1.23 to −0.32), while non-HDL-C showed a moderate effect (*d* = 0.50, 95% CI: 0.06–0.95). The between-group difference in fasting glucose (GLU 0′) was small to moderate, with the 95% CI including zero (*d* = 0.42, 95% CI: −0.02–0.86).

### 2.3. Hormonal Parameters

Hormonal profiles were broadly comparable between women with and without central obesity (Table 3). Luteinizing hormone (LH), follicle-stimulating hormone (FSH), and the LH/FSH ratio showed no significant differences between groups (*p* > 0.05). Estradiol (E2), total testosterone (T), androstenedione (A4), and dehydroepiandrosterone sulfate (DHEA-S) levels were also similar. Sex hormone-binding globulin (SHBG) tended to be lower in centrally obese women, but the difference was not significant (*p* = 0.313). Overall, no substantial hormonal differences were observed, suggesting that the observed metabolic and vascular differences were not accompanied by substantial differences in gonadotropic or androgenic hormone profiles.

### 2.4. Inflammatory and Endothelial Markers; Leukocyte and Platelet Counts

The significantly higher sE-selectin concentrations observed in women with WHtR > 0.50 were associated with a large between-group effect (Cohen’s *d* = 1.45), with a mean difference of 31.23 ng/mL (95% CI: 21.57–40.89). The remaining inflammatory, leukocyte, and platelet parameters showed small-to-moderate effect sizes, with 95% CIs crossing zero, consistent with the absence of statistically significant between-group differences (Table 4).

### 2.5. Correlations with Metabolic Parameters

**Figure 1:**
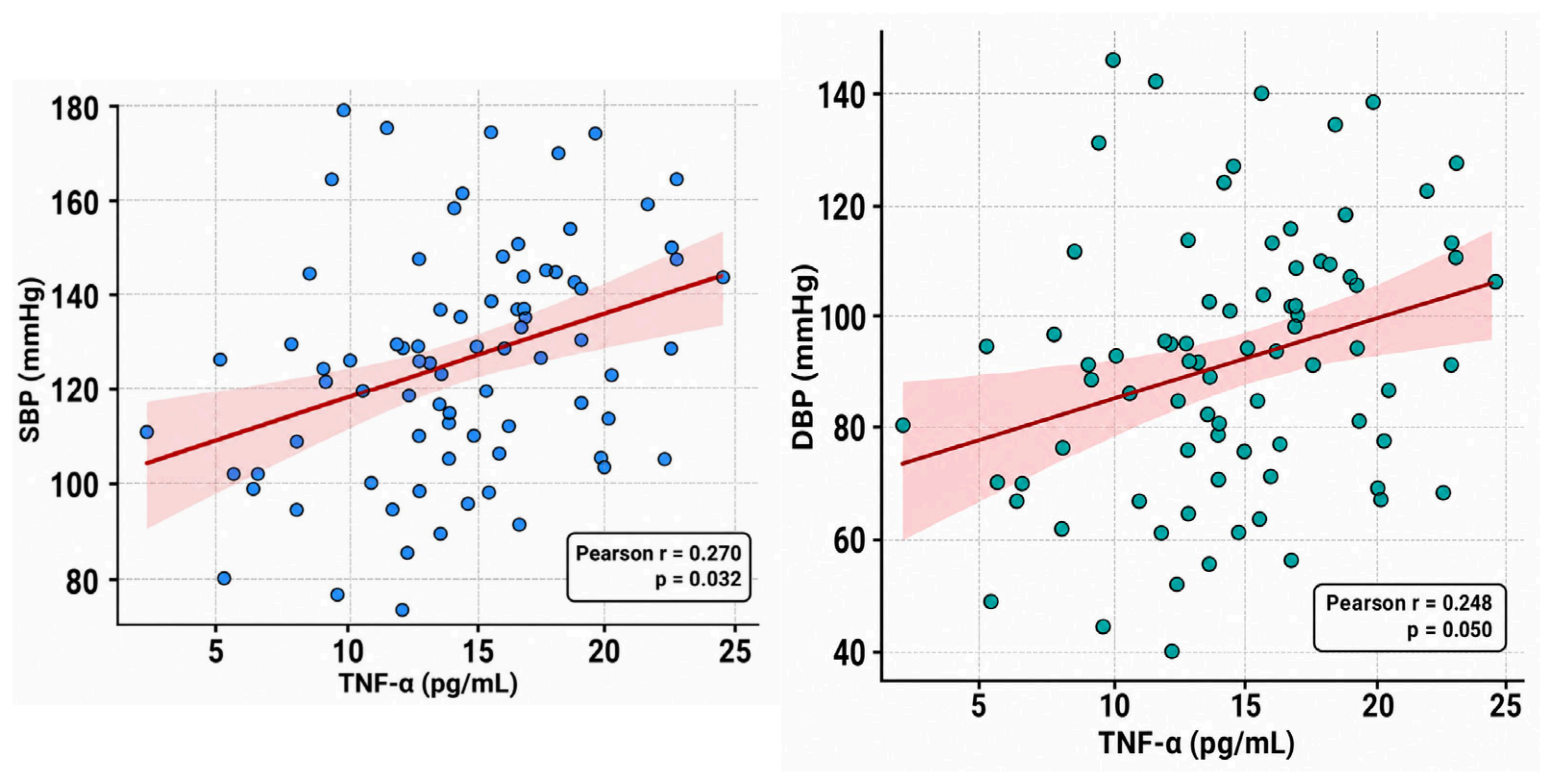
Positive Relationship of TNF-α with Systolic Blood Pressure (SBP) and Diastolic Blood Pressure (DBP) in Women with PCOS.

**Figure 2:**
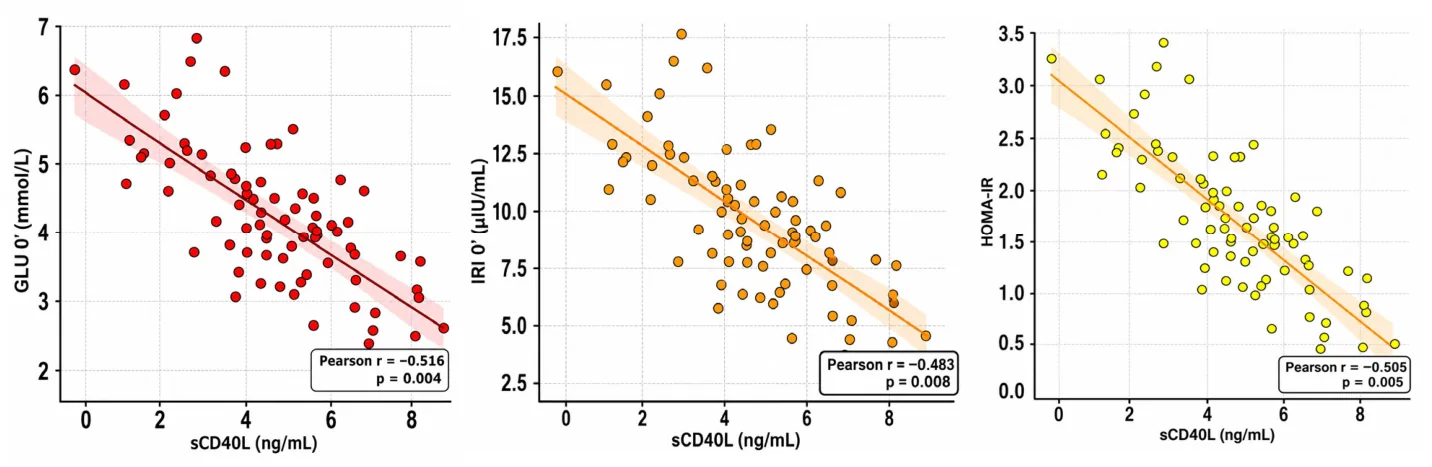
Negative Associations of sCD40L with GLU 0’, IRI 0’ and HOMA-IR in Women with PCOS.

**Figure 3:**
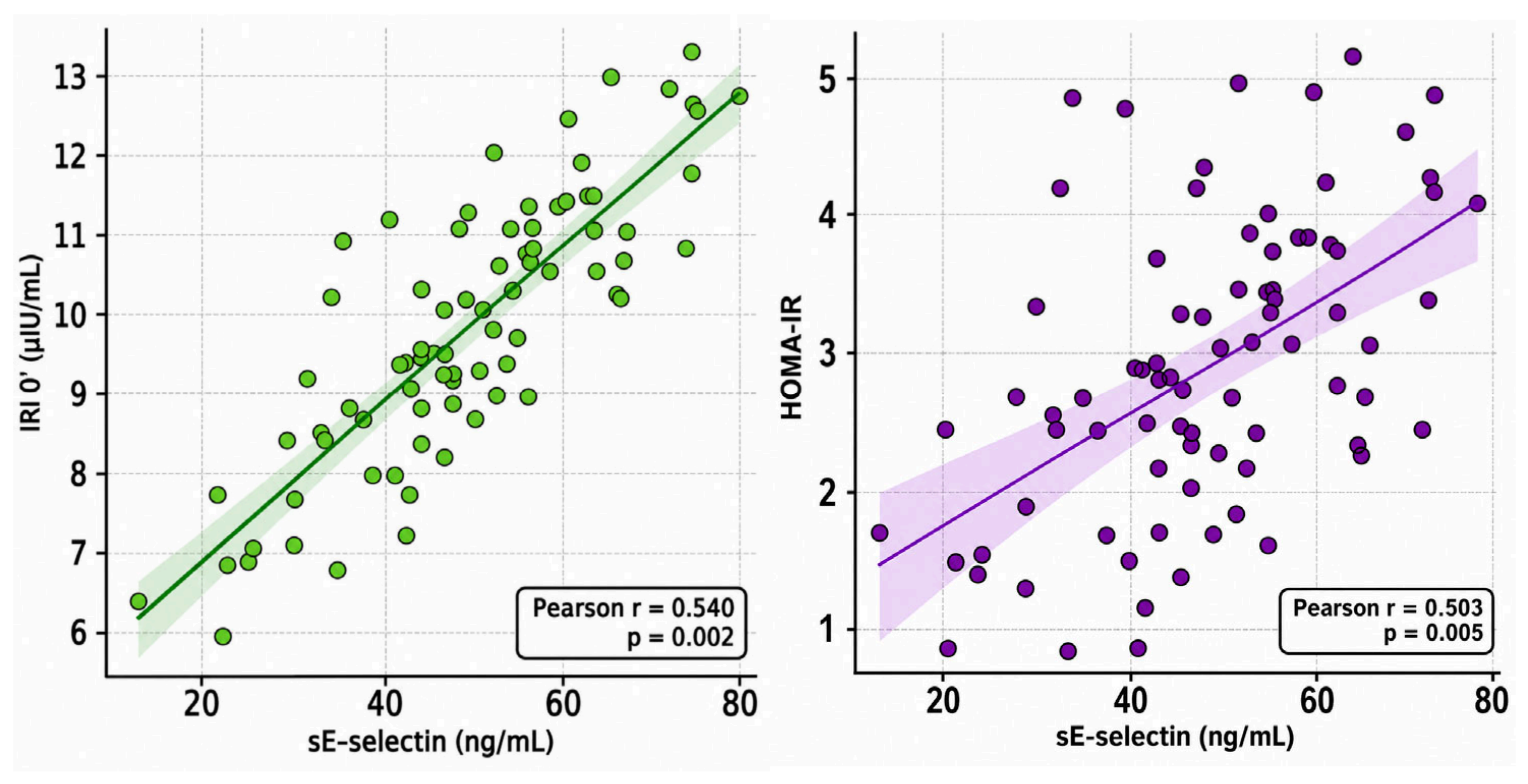
Positive Correlations of sE-selectin with IRI 0′and HOMA-IR in Women with PCOS.

In the study cohort, TNF-α correlated positively with SBP (r = 0.270, 95% CI: 0.02 to 0.48, *p* = 0.032) and DBP (r = 0.248, 95% CI: 0.00 to 0.46, *p* = 0.050) (Figure 1). sCD40L showed inverse correlations with GLU 0′ (r = −0.516, 95% CI: −0.74 to −0.18, *p* = 0.004), IRI 0′ (r = −0.483, 95% CI: −0.72 to −0.14, *p* = 0.008), and HOMA-IR (r = −0.505, 95% CI: −0.73 to −0.17, *p* = 0.005) (Figure 2). In contrast, sE-selectin correlated positively with IRI 0′ (r = 0.540, 95% CI: 0.21 to 0.75, *p* = 0.002) and HOMA-IR (r = 0.503, 95% CI: 0.16 to 0.73, *p* = 0.005) (Figure 3). These correlations should be interpreted in the context of the cross-sectional design and therefore do not imply causality.

### 2.6. Multivariable Regression Analyses

To identify independent determinants of circulating biomarker concentrations, multivariable linear regression analyses were performed after adjustment for age, BMI, and waist circumference. GLU 0′ remained independently and inversely associated with sCD40L concentrations (β = −0.27, 95% CI: −0.50 to −0.04, *p* = 0.020), whereas IRI 0′ emerged as the strongest independent predictor of sE-selectin levels (β = 0.41, 95% CI: 0.17 to 0.65, *p* < 0.001). Neither TNF-α nor IL-6 showed an independent association with sCD40L or sE-selectin (Table 5).

## 3. Discussion

The present study investigated the relationships between endothelial and immune activation markers, insulin resistance, and central obesity in women with PCOS. Women with elevated WHtR exhibited a less favorable cardiometabolic profile characterized by higher IRI 0′, HOMA-IR, TG, non-HDL-C, and SBP, together with lower HDL-C. These findings are consistent with previous evidence indicating that visceral adiposity is a major determinant of metabolic risk in PCOS [21,22,23], while anthropometric indices reflecting abdominal adiposity may provide clinically relevant information beyond BMI alone [24,25]. The absence of significant differences in gonadotropic and androgenic hormone concentrations between groups further suggests that the observed metabolic and vascular alterations were primarily associated with adiposity-related metabolic disturbances rather than endocrine differences.

A major finding of this study was the association between sE-selectin and insulin resistance. Women with central obesity exhibited significantly higher circulating sE-selectin concentrations, while fasting insulin emerged as the strongest independent predictor of sE-selectin levels after adjustment for age, BMI, and waist circumference. These observations are consistent with previous reports demonstrating increased circulating sE-selectin concentrations in obesity and metabolic disorders [17,18,26]. In women with PCOS, insulin resistance has been associated with oxidative stress, enhanced leukocyte adhesion, and endothelial activation, providing a potential mechanistic link between metabolic dysfunction and vascular alterations [27,28]. Experimental and clinical studies suggest that insulin resistance and compensatory hyperinsulinemia may contribute to endothelial activation through mechanisms involving oxidative stress, impaired endothelial insulin signaling, and inflammatory pathways, leading to altered leukocyte–endothelial interactions [29,30,31]. Collectively, these findings support the concept that endothelial activation represents an early vascular manifestation of metabolic dysfunction in PCOS. In obesity and PCOS, inflammatory signaling may be compartmentalized within adipose tissue and may involve local macrophage activation, altered adipokine secretion, oxidative stress, and endothelial-specific pathways. Consequently, endothelial activation may become detectable through circulating endothelial markers such as sE-selectin even when circulating concentrations of selected inflammatory cytokines remain comparable between groups. This interpretation is consistent with the present finding that sE-selectin, but not TNF-α or IL-6, differed significantly according to central adiposity.

Interestingly, circulating TNF-α and IL-6 concentrations did not differ significantly between groups and were not independently associated with either sE-selectin or sCD40L. This observation is consistent with previous evidence showing that circulating inflammatory cytokines may display heterogeneous associations with metabolic and vascular alterations in PCOS [32]. This apparent dissociation suggests that endothelial activation in PCOS may precede detectable elevations in circulating inflammatory cytokines. Local adipose tissue inflammation, oxidative stress, and endothelial-specific signaling pathways may therefore contribute to endothelial dysfunction even when systemic inflammatory markers remain within comparable ranges.

The absence of significant differences in circulating TNF-α and IL-6 despite the marked differences in BMI and central adiposity deserves consideration. Although obesity and PCOS are frequently associated with low-grade inflammation, circulating concentrations of individual cytokines may not necessarily parallel the degree of adiposity or reflect tissue-specific inflammatory activity. Systemic concentrations of TNF-α and IL-6 may be influenced by multiple metabolic and physiological factors and may not adequately capture inflammatory signaling within visceral adipose tissue or at the endothelial level. Moreover, the relatively young age of our participants and the absence of overt cardiometabolic disease may have contributed to the lack of detectable differences in circulating cytokines. Thus, our findings should not be interpreted as evidence for the absence of inflammation in PCOS, but rather as an indication that circulating TNF-α and IL-6 did not discriminate between the two adiposity-related phenotypes in the present cohort.

Unlike sE-selectin, sCD40L demonstrated inverse associations with fasting glucose, fasting insulin, and HOMA-IR. This finding differs from several studies reporting elevated sCD40L concentrations in obesity, IR, and PCOS [33,34,35,36]. However, the available evidence remains heterogeneous. Although increased circulating sCD40L concentrations have frequently been described in obesity and PCOS, accumulating evidence suggests that platelet activation, insulin signaling, and the metabolic milieu collectively influence sCD40L release, indicating that its relationship with IR may be context-dependent rather than uniformly linear. Supporting this concept, Gateva et al. reported that circulating sCD40L was associated with IR, but not with glucose tolerance, in obese nondiabetic individuals [37]. Furthermore, experimental studies have shown that acute hyperglycemia and euglycemic hyperinsulinemia may transiently suppress circulating sCD40L concentrations [38]. In addition, acute hyperinsulinemia has been shown to modulate several prothrombotic and vascular biomarkers, supporting the concept that insulin exerts direct effects on vascular homeostasis beyond glucose regulation [39]. Together, these observations suggest that circulating sCD40L may reflect dynamic metabolic and platelet-related responses rather than a simple linear consequence of worsening IR. Consistent with this interpretation, fasting glucose remained independently and inversely associated with sCD40L concentrations in the present study after adjustment for potential confounding factors.

Activated platelets are the principal source of circulating sCD40L, and CD40/CD40L signaling plays an important role in inflammatory and thrombotic processes that may be influenced by metabolic stress [40,41]. One possible explanation for the observed inverse associations is altered platelet activation or changes in sCD40L release under conditions of chronic metabolic stress. Although this hypothesis remains speculative, it may partly account for the heterogeneous findings reported across different metabolic populations and warrants further mechanistic investigation.

Our findings further emphasize the importance of central adiposity in shaping the cardiometabolic phenotype of PCOS. Women with increased WHtR exhibited a cluster of metabolic abnormalities, including hyperinsulinemia, dyslipidemia, and elevated systolic blood pressure, supporting previous evidence that visceral fat accumulation contributes to insulin resistance and endothelial dysfunction through mechanisms involving increased free fatty acid release, oxidative stress, and adipokine dysregulation [20,42]. Furthermore, anthropometric indices reflecting abdominal adiposity, such as WHtR and waist circumference, have been shown to provide better discrimination of cardiometabolic risk than BMI in women with PCOS [24,25]. Consistent with these observations, our results suggest that central obesity and insulin resistance, rather than differences in androgen status, are major determinants of metabolic and vascular alterations in PCOS [43].

From a clinical perspective, fasting insulin emerged as the principal independent determinant of circulating sE-selectin concentrations, emphasizing the close relationship between insulin resistance and endothelial activation in PCOS. The complementary behavior of sE-selectin and sCD40L suggests that endothelial and immune-related biomarkers may reflect distinct biological pathways involved in cardiometabolic risk. Clinically, sE-selectin may reflect endothelial activation, whereas sCD40L may indicate immune- and platelet-related activation. Although these biomarkers may help characterize early vascular and cardiometabolic alterations in women with PCOS, they remain investigational rather than routine clinical tests, and their potential value for cardiovascular risk stratification, prognostication, and therapeutic decision-making requires validation in prospective longitudinal studies.

The strengths of this study include the use of WHtR as a marker of central obesity, comprehensive metabolic and hormonal characterization, simultaneous assessment of endothelial and immune biomarkers, and adjustment for major anthropometric confounders in multivariable analyses. Several limitations should also be acknowledged. First, the cross-sectional design precludes causal inference. Second, the moderate sample size may have limited the statistical power to detect weaker associations. Third, the absence of a non-PCOS control group prevents discrimination between alterations specifically related to PCOS and those attributable to obesity or insulin resistance. Another limitation is the absence of a broader inflammatory, endothelial, and adipokine panel, including CRP, MCP-1, ICAM-1, VCAM-1, adiponectin, leptin, and other adipose-tissue-derived mediators, which could have provided a more comprehensive characterization of the inflammatory–vascular phenotype in PCOS.

In conclusion, women with PCOS and central obesity exhibited a more adverse metabolic profile together with higher circulating sE-selectin concentrations, supporting a close relationship between insulin resistance and endothelial activation. In contrast, sCD40L demonstrated inverse associations with indices of insulin resistance, suggesting more complex regulation of immune–vascular signaling pathways. These findings emphasize the heterogeneous mechanisms linking central adiposity, metabolic dysfunction, and vascular alterations in PCOS and support further investigation of circulating endothelial and immune biomarkers as potential tools for early cardiometabolic risk assessment.

## 4. Materials and Methods

### 4.1. Study Design and Participants

This cross-sectional study included 80 women with polycystic ovary syndrome (PCOS), aged 18–35 years, who were enrolled at the Clinic of Endocrinology and Metabolic Diseases, “Sv. Georgy” University Hospital of Plovdiv, from June 2020 to June 2023. The study was designed as an internal comparison between women with PCOS stratified according to central obesity status; no non-PCOS control group was included. All participants were diagnosed with PCOS according to the Rotterdam criteria [44], requiring at least two of the following: (i) oligo/anovulation, (ii) clinical and/or biochemical hyperandrogenism, and (iii) polycystic ovarian morphology on ultrasound, after exclusion of related disorders (e.g., congenital adrenal hyperplasia, Cushing’s syndrome, thyroid dysfunction, or hyperprolactinemia). Furthermore, individuals with diabetes mellitus, chronic inflammatory or autoimmune conditions, or those using insulin-sensitizing or lipid-lowering medications (such as metformin or statins), hormonal contraceptives, or currently pregnant were excluded. All women in the final study population were non-smokers.

Participants were stratified according to central obesity using the waist-to-height ratio (WHtR), applying the established cut-off value of 0.50 [19]. Accordingly, 40 women were classified as having central obesity (WHtR > 0.50) and 40 as not having central obesity (WHtR ≤ 0.50).

All clinical, biochemical, and hormonal assessments were performed during the early follicular phase of the menstrual cycle (days 2–5) or following progesterone-induced withdrawal bleeding in women with oligo- or amenorrhea.

The study protocol was approved by the institutional Ethics Committee of Medical University of Plovdiv, Bulgaria, and all procedures adhered to the Declaration of Helsinki (2013 revision). Written informed consent was obtained from all participants before enrollment.

### 4.2. Anthropometric and Clinical Assessment

Body weight and height were measured using standardized calibrated instruments, with participants wearing light clothing and no shoes. Body mass index (BMI) was calculated as weight (kg) divided by height squared (m^2^). Waist circumference (W) was measured midway between the lower rib margin and the iliac crest, and hip circumference (H) at the widest point over the buttocks. The waist-to-hip ratio (WHR) and waist-to-height ratio (WHtR) were calculated accordingly.

Blood pressure was measured twice in the seated position after a 10 min rest using a validated automatic sphygmomanometer, and the mean of two readings was recorded for both systolic (SBP) and diastolic (DBP) pressure.

### 4.3. Biochemical Analysis; Leukocyte and Platelet Counts

Fasting plasma glucose was measured using the glucose oxidase–peroxidase (GOD–POD) method, and fasting serum insulin concentrations were determined by chemiluminescent immunoassay (CLIA; Beckman Coulter, Brea, CA, USA). The analytical sensitivity of the insulin assay was 0.03 μIU/mL, with intra- and inter-assay coefficients of variation below 6%. Insulin resistance was estimated using the homeostasis model assessment for insulin resistance (HOMA-IR), calculated as: HOMA-IR = fasting glucose (mmol/L) × fasting insulin (μIU/mL)/22.5.

Serum total cholesterol (TC), triglycerides (TG), and high-density lipoprotein cholesterol (HDL-C) were determined by standard enzymatic methods using commercial reagents (Schneiders Medizintechnik, Zwolle, The Netherlands) on a Delta Kone automated analyzer (Kone Instruments, Espoo, Finland). Low-density lipoprotein cholesterol (LDL-C) was calculated using the Friedewald equation, and non-HDL cholesterol was calculated as total cholesterol minus HDL cholesterol.

Leukocyte (WBC) and platelet (PLT) counts were determined using an automated hematology analyzer based on the Coulter principle. Samples showing abnormal cell morphology or platelet aggregation were verified manually.

### 4.4. Hormonal Assays

Circulating concentrations of reproductive hormones and androgen-related parameters were assessed by automated chemiluminescent immunoassays (CLIA) using commercially available platforms and reagents, following the manufacturers’ validated protocols. Serum LH and FSH levels were determined using sandwich-type immunochemical assays (Beckman Coulter, Inc., Brea, CA, USA), with analytical sensitivities of 0.2 IU/L and 0.2 mIU/mL, respectively. Estradiol (E2) concentrations were obtained using a competitive immunochemical CLIA method (Beckman Coulter, Inc., Clare, Ireland), with an assay sensitivity of 73 pmol/L.

Total testosterone (T) and DHEA-S were analyzed using CLIA-based assays provided by Beckman Coulter, Inc. (Brea, CA, USA). The reported analytical sensitivities were 0.35 ng/mL for T and <2 μg/dL for DHEA-S. SHBG measurements were performed using the Access 2 Immunoassay System (Beckman Coulter, Inc., Brea, CA, USA), with a sensitivity threshold of 0.33 nmol/L and an analytical measuring range extending to 180 nmol/L. Androstenedione (A4) levels were determined by a CLIA assay (catalog no. L2KAO2; Siemens Healthcare Diagnostics, Tarrytown, NY, USA), with an analytical sensitivity of 1.0 nmol/L.

The analytical reliability of the assays was evaluated according to the manufacturers’ quality specifications. Intra- and inter-assay coefficients of variation (CVs) were generally below 10%; however, higher variability was observed for E2 and A4 measurements, consistent with the reported assay performance characteristics. Internal quality controls, calibration procedures, and assay validation were performed throughout the analytical process in accordance with the manufacturers’ recommendations.

All hormonal assessments were performed during the early follicular phase of the menstrual cycle.

### 4.5. Inflammatory and Endothelial Markers

Serum tumor necrosis factor-α (TNF-α) and interleukin-6 (IL-6) concentrations were determined using commercially available enzyme-linked immunosorbent assay (ELISA) kits (DRG Instruments GmbH, Marburg, Germany) according to the manufacturers’ instructions. The analytical sensitivities were 3.0 pg/mL for TNF-α and 2.0 pg/mL for IL-6. The intra-assay coefficient of variation for the IL-6 assay was <7.7%.

Serum soluble CD40 ligand (sCD40L) and soluble E-selectin (sE-selectin) concentrations were measured using commercially available ELISA kits (Bender MedSystems, Vienna, Austria) following the manufacturers’ protocols. Both assays demonstrated acceptable analytical performance, with intra-assay coefficients of variation < 8% and inter-assay coefficients of variation < 10%.

### 4.6. Statistical Analysis

Statistical analyses were performed using IBM SPSS Statistics for Windows, version 21.0 (IBM Corp., Armonk, NY, USA). Continuous variables are presented as mean ± standard deviation (SD). Data normality was assessed using the Shapiro–Wilk test.

Comparisons between women with and without central obesity (WHtR > 0.50 vs. ≤0.50) were performed using the independent-samples Student’s *t*-test. Pearson’s correlation coefficient (r) was used to assess associations between continuous variables. Correlation analyses were performed using available paired observations for each pair of variables.

To complement hypothesis testing, effect sizes with corresponding 95% confidence intervals (CIs) were calculated for the main metabolic findings. For continuous variables, standardized between-group effect sizes (Cohen’s *d*, with Hedges’ *g* applied where appropriate) and their 95% CIs were estimated to quantify the magnitude and precision of the observed differences between women with WHtR ≤ 0.50 and WHtR > 0.50.

Multivariable linear regression analyses were performed after adjustment for age, BMI, and waist circumference. Standardized regression coefficients (β) with corresponding 95% confidence intervals (CIs) and *p*-values were reported to quantify the strength, direction, and precision of independent associations.

No correction for multiple comparisons was applied because the analyses were based on a limited number of pre-specified hypotheses. All statistical tests were two-sided, and *p* < 0.05 was considered statistically significant.

## 5. Conclusions

In conclusion, this study provides evidence that central adiposity in women with PCOS is associated with a distinct cardiometabolic and vascular phenotype characterized by marked insulin resistance and increased circulating sE-selectin, whereas circulating TNF-α and IL-6 did not differ significantly between the two groups. The divergent associations of sCD40L and sE-selectin with metabolic parameters further support the concept that endothelial and immune/platelet activation may represent partially distinct biological pathways in PCOS.

The novelty of the present findings lies in the identification of a differential relationship between central adiposity, metabolic disturbances, and circulating markers of endothelial and immune/platelet activation within the PCOS population. Biologically, these findings suggest that endothelial activation may be detectable even in the absence of differences in selected circulating inflammatory cytokines, highlighting the complex and potentially compartmentalized nature of inflammation and vascular dysfunction in PCOS.

From a translational perspective, combined assessment of metabolic status and complementary vascular and immune biomarkers may contribute to a more refined characterization of cardiometabolic phenotypes in women with PCOS. However, sE-selectin and sCD40L should currently be considered investigational biomarkers rather than routine clinical tools. Therapeutically, the observed association between central adiposity, insulin resistance, and endothelial activation supports the importance of strategies targeting adiposity and metabolic dysfunction as potentially modifiable components of early vascular risk. Prospective studies incorporating broader inflammatory and adipokine panels and long-term cardiovascular outcomes are needed to establish the prognostic value and potential clinical utility of these biomarkers.

## Figures and Tables

**Table 1:** Age, anthropometric and clinical characteristics of women with PCOS according to central adiposity. Women were stratified according to WHtR (≤0.50, *n* = 40; >0.50, *n* = 40). Data are presented as mean ± SD. Effect sizes and corresponding 95% confidence intervals for between-group differences are reported in the Results.

Parameter	PCOS with WHtR ≤ 0.50 (*n* = 40)	PCOS with WHtR > 0.50 (*n* = 40)
Age (years)	23.10 ± 4.05	25.20 ± 5.44 NS
Height (cm)	166.44 ± 7.55	166.37 ± 5.48 NS
Weight (kg)	59.66 ± 9.62	85.52 ± 11.26 ***
BMI (kg/m^2^)	21.46 ± 2.60	30.88 ± 3.80 ***
Waist (cm)	70.26 ± 6.38	97.09 ± 7.79 ***
Hip (cm)	94.87 ± 7.35	111.49 ± 7.98 **
WHR	0.74 ± 0.06	0.87 ± 0.06 ***
WHtR	0.42 ± 0.04	0.58 ± 0.05 ***
SBP (mmHg)	110.64 ± 11.71	118.68 ± 11.50 **
DBP (mmHg)	71.15 ± 8.39	75.15 ± 10.33 NS

NS—not significant (*p* > 0.05); **—*p* < 0.01; ***—*p* < 0.001.

**Table 2:** Metabolic and atherogenic parameters in the two studied groups of women with PCOS. Women were stratified according to WHtR (≤0.50, *n* = 40; >0.50, *n* = 40). Data are presented as mean ± SD. Effect sizes and corresponding 95% confidence intervals for between-group differences are reported in the Results.

Parameter	PCOS with WHtR ≤ 0.50 (*n* = 40)	PCOS with WHtR > 0.50 (*n* = 40)
GLU 0′ (mmol/L)	4.73 ± 0.45	4.99 ± 0.75 NS
IRI 0′ (μIU/mL)	6.56 ± 3.09	11.39 ± 5.87 ***
HOMA-IR	1.41 ± 0.76	2.64 ± 1.68 ***
TC (mmol/L)	4.36 ± 0.97	4.57 ± 0.78 NS
LDL-C (mmol/L)	2.60 ± 0.99	2.85 ± 0.73 NS
HDL-C (mmol/L)	1.47 ± 0.49	1.17 ± 0.25 **
TG (mmol/L)	0.75 ± 0.32	1.22 ± 0.49 ***
Non-HDL-C	2.95 ± 1.01	3.40 ± 0.77 *

NS—not significant (*p* > 0.05); *—*p* < 0.05; **—*p* < 0.01; ***—*p* < 0.001.

**Table 3:** Hormonal parameters in the two studied groups of women with PCOS. Women were stratified according to WHtR (≤0.50, *n* = 40; >0.50, *n* = 40). Data are presented as mean ± SD.

Parameter	PCOS with WHtR ≤ 0.50 (*n* = 40)	PCOS with WHtR > 0.50 (*n* = 40)
LH (IU/L)	7.91 ± 3.33	8.59 ± 4.34 NS
FSH (mIU/mL)	5.78 ± 1.78	5.43 ± 1.92 NS
LH/FSH	1.38 ± 0.76	1.88 ± 1.40 NS
E2 (pg/mL)	251.31 ± 99.11	244.56 ± 101.11 NS
Total testosterone (ng/mL)	0.67 ± 0.17	0.70 ± 0.21 NS
Androstenedione (ng/mL)	3.84 ± 1.06	3.77 ± 1.66 NS
DHEA-S (μg/dL)	285.85 ± 101.62	278.52 ± 104.37 NS
SHBG (nmol/L)	44.50 ± 20.01	35.04 ± 18.43 NS

NS—not significant (*p* > 0.05).

**Table 4:** Inflammatory and endothelial markers; leukocyte and platelet counts in the two studied groups of women. Women were stratified according to WHtR (≤0.50, *n* = 40; >0.50, *n* = 40). Data are presented as mean ± SD. Effect sizes and corresponding 95% confidence intervals for between-group differences are reported in the Results.

Parameter	PCOS with WHtR ≤ 0.50 (*n* = 40)	PCOS with WHtR > 0.50 (*n* = 40)
TNF-α (pg/mL)	7.43 ± 4.50	8.39 ± 5.28 NS
IL-6 (pg/mL)	1.45± 0.91	1.36 ± 0.88 NS
sCD40L (ng/mL)	10.37 ± 4.17	9.43 ± 4.29 NS
sE-selectin (ng/mL)	41.70 ± 18.06	72.93 ± 24.64 **
Leucocytes (×10^9^/L)	6.11± 1.72	6.78 ± 1.27 NS
PLT (×10^9^/L)	271.11± 67.24	294.85 ± 78.32 NS

NS—not significant (*p* > 0.05); **—*p* < 0.01.

**Table 5:** Independent predictors of circulating sCD40L and sE-selectin concentrations in women with PCOS (multivariable linear regression analysis).

Dependent Variable	Predictor	Standardized β	95% CI	*p*
sCD40L (ng/mL)	GLU 0′ (mmol/L) IRI 0′ (μIU/mL) TNF-α (pg/mL) IL-6 (pg/mL)	−0.27 −0.18 0.09 −0.07	−0.50 to −0.04 −0.41 to 0.05 −0.13 to 0.31 −0.28 to 0.14	0.020 0.120 0.420 0.510
sE-selectin (ng/mL)	GLU 0′ (mmol/L) IRI 0′ (μIU/mL) TNF-α (pg/mL) IL-6 (pg/mL)	0.10 0.41 0.12 0.08	−0.11 to 0.31 0.17 to 0.65 −0.09 to 0.33 −0.13 to 0.29	0.340 <0.001 0.270 0.450

Models were adjusted for age, BMI, and waist circumference. Standardized regression coefficients (β), 95% confidence interval (CI) and corresponding *p*-values are presented.

## Data Availability

The data that support the findings of this study are available from the corresponding author upon reasonable request. Due to ethical restrictions and participant confidentiality, the dataset is not publicly available.

## Abbreviations

The following abbreviations are used in this manuscript:

PCOS	Polycystic Ovary Syndrome
IR	Insulin Resistance
BMI	Body Mass Index
WHR	Waist-to-Hip Ratio
WHtR	Waist-to-Height Ratio
HOMA-IR	Homeostatic Model Assessment for Insulin Resistance
SBP	Systolic Blood Pressure
DBP	Diastolic Blood Pressure
GLU 0′	Fasting Glucose
IRI 0′	Fasting Insulin
TC	Total Cholesterol
HDL-C	High-Density Lipoprotein Cholesterol
LDL-C	Low-Density Lipoprotein Cholesterol
TG	Triglycerides
LH	Luteinizing Hormone
FSH	Follicle-Stimulating Hormone
E2	Estradiol
SHBG	Sex Hormone-Binding Globulin
A4	Androstenedione
DHEA-S	Dehydroepiandrosterone Sulfate
sCD40L	Soluble CD40 Ligand
IL-6	Interleukin 6
TNF- α	Tumor Necrosis Factor alpha
WBC	Leukocytes
PLT	Platelets
ELISA	Enzyme-Linked Immunosorbent Assay
